# In Vitro Models for the Study of Liver Biology and Diseases: Advances and Limitations

**DOI:** 10.1016/j.jcmgh.2022.11.008

**Published:** 2022-11-26

**Authors:** Savneet Kaur, Srivatsan Kidambi, Martí Ortega-Ribera, Le Thi Thanh Thuy, Natalia Nieto, Victoria C. Cogger, Wei-Fen Xie, Frank Tacke, Jordi Gracia-Sancho

**Affiliations:** 1 Department of Molecular and Cellular Medicine, Institute of Liver and Biliary Sciences, New Delhi, India; 2 Department of Chemical and Biomolecular Engineering, University of Nebraska, Lincoln, Nebraska; 3 Department of Medicine, Beth Israel Deaconess Medical Center, Harvard Medical School, Boston, Massachusetts; 4 Department of Hepatology, Graduate School of Medicine, Osaka Metropolitan University, Osaka, Japan; 5 Department of Pathology, University of Illinois at Chicago, Chicago, Illinois; 6 Faculty of Medicine and Health, University of Sydney, Sydney, Australia; 7 Department of Gastroenterology, Changzheng Hospital, Naval Medical University, Shanghai, China; 8 Department of Hepatology and Gastroenterology, Charité Universitätsmedizin Berlin, Berlin, Germany; 9 Liver Vascular Biology, IDIBAPS Biomedical Research Institute, CIBEREHD, Barcelona, Spain; 10 Department of Visceral Surgery and Medicine, Inselspital, Bern University Hospital, University of Bern, Switzerland; 11 Department for BioMedical Research, Visceral Surgery and Medicine, University of Bern, Switzerland

**Keywords:** Hepatic Sinusoid, Mechanobiology, Omics, Bioengineering, Cirrhosis, NAFLD, NASH, CLD, chronic liver disease, EC, endothelial cell, ECM, extracellular matrix, HBV, hepatitis B virus, HCC, hepatocellular carcinoma, HSC, hepatic stellate cell, LoC, liver-on-a-chip, LSEC, liver sinusoidal endothelial cell, NAFLD, nonalcoholic fatty liver disease, NASH, nonalcoholic steatohepatitis, PCLS, precision cut liver slices, PDGF, platelet-derived growth factor, PNPLA3, patatin-like phospholipase domain containing 3, sc-RNA Seq, single-cell RNA sequencing, snRNA-seq, single-nuclei RNA sequencing, 2D, 2-dimensional, 3D, 3-dimensional

## Abstract

In vitro models of liver (patho)physiology, new technologies, and experimental approaches are progressing rapidly. Based on cell lines, induced pluripotent stem cells or primary cells derived from mouse or human liver as well as whole tissue (slices), such in vitro single- and multicellular models, including complex microfluidic organ-on-a-chip systems, provide tools to functionally understand mechanisms of liver health and disease. The International Society of Hepatic Sinusoidal Research (ISHSR) commissioned this working group to review the currently available in vitro liver models and describe the advantages and disadvantages of each in the context of evaluating their use for the study of liver functionality, disease modeling, therapeutic discovery, and clinical applicability.

**Summary**

This review provides a glimpse on the most advanced methods for studying liver sinusoidal biology in vitro to uncover liver disease pathophysiology focusing on liver-on-a-chip and microfluidic devices, liver scaffolds and matrices, mechanobiological cues, spheroids/organoids, liver slices and omics.

Liver disease represents one of the leading causes of death worldwide, and the incidence of some pathologies, such as nonalcoholic fatty liver disease (NAFLD), nonalcoholic steatohepatitis (NASH), and liver cancer, continues to increase.^1^ Despite years of research, liver diseases still have limited treatment options in the clinic. This paucity of treatments is explained partly by the limitations of traditional in vitro tools and animal models that do not accurately mimic the clinical pathophysiology of diseases and have a low accuracy for drug discovery purposes. Indeed, several studies have shown that traditional cell culture methodologies do not reflect the complexity of a human liver in vivo and thus cannot predict drug sensitivity. In contrast, animal models differ in biology compared with human pathologies, which explains why promising therapies tested in animal models often fail when tested in human beings and, unfortunately, the field of hepatology has numerous recent examples of failures in clinical phases.^2^ With the advent of precision medicine, which offers much hope for individual patient outcomes, there is increased demand for robust and patient-specific tools to better improve our understanding and treatment of complex and multifactorial diseases such as liver diseases. Advances in vascular biology, microfluidics, and bioengineering have led to the development of sophisticated in vitro models that could fill this gap (Figure 1). In addition, omics techniques provide further insight to preclinical research in hepatology. In this review, we discuss the benefits and limitations of advanced in vitro research techniques that presently are being applied to the study of liver diseases and further critique how these tools may provide insight into the prediction of patient responses to a therapy.

**Figure 1:**
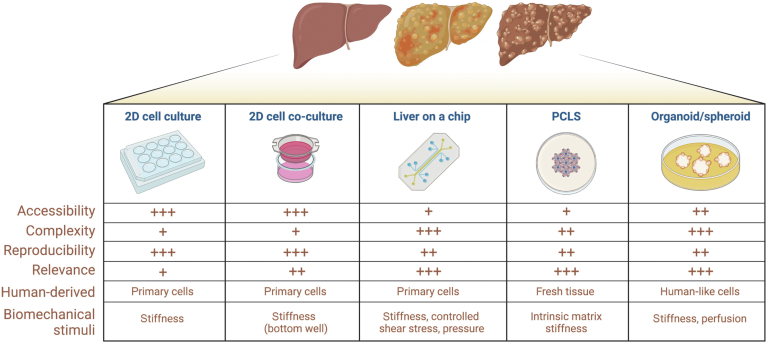
**Schematic view of mostly used in vitro models in hepatology.** +, low; ++, medium; +++, high.

## Liver-on-a-Chip and Microfluidic Devices

During the 21st century, the development of biology-inspired devices aimed at mimicking the sinusoidal niche integrating microfluidics led to the rapidly evolving liver-on-a-chip (LoC) technology.^3^ The design of these in vitro liver-resembling tools, which have been reviewed extensively by Ortega-Ribera et al^4^ and is out of the scope of the present review, is inspired in sinusoidal cell biology, architecture, and hemodynamics, but materialized under each research teams’ eyes in terms of appearance, size, fabrication procedures, costs, and microfluidics integration, leading to significant variation in the finalized product.

**Figure 2:**
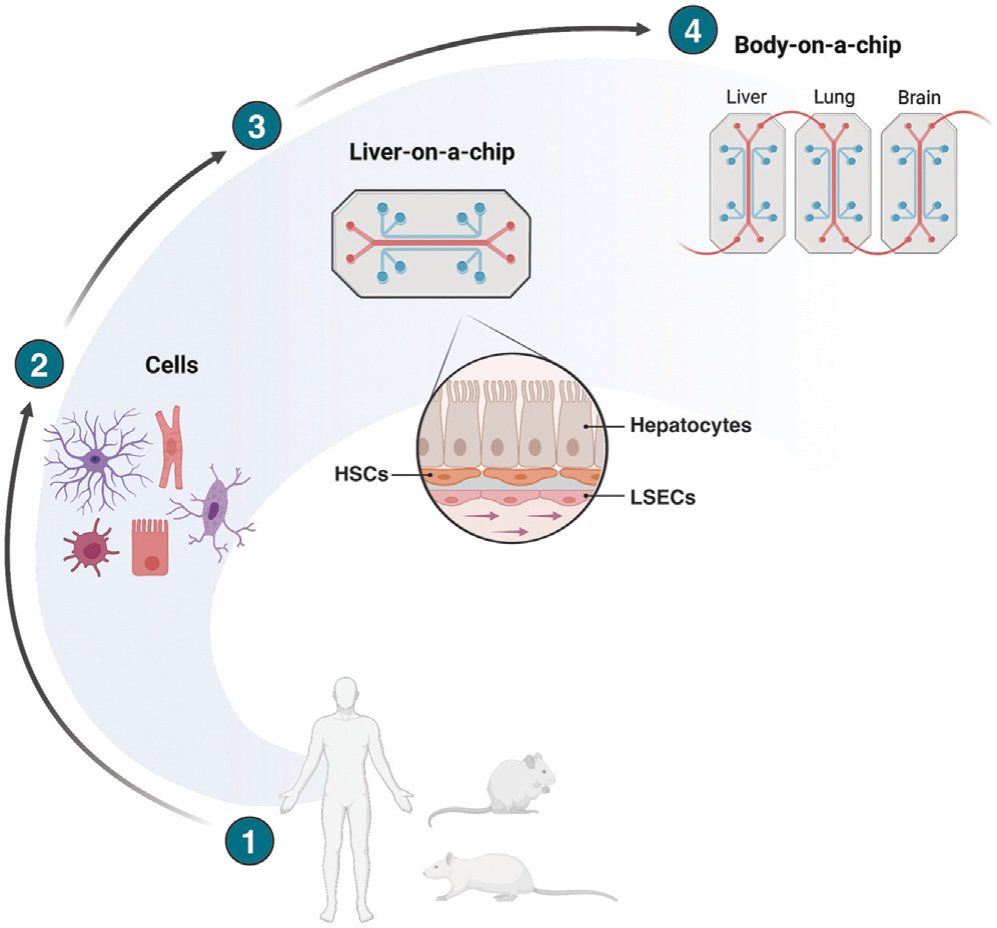
**Fromsingle cells to liver-on-a-chip and body on-a-chip.**

The latest advances in the field include chronic liver disease–specific devices, LoC models designed to study key pathophysiological processes in the development of liver disease, and to understand the interconnection with other organs-on-chip to better depict liver functions and systemic implications (Figure 2). Multi-organ chips, for instance, liver-, adipose-, and gut-on-a-chip connected, may be particularly suitable to understand organ-crosstalk in chronic liver disease (CLD), such as NAFLD/NASH or cholangiopathies.^5^

In recent years, disease-focused LoC devices mimicking some of the landmark etiological characteristics of CLD have been developed. Fat accumulation in hepatocytes, occurring in NAFLD/NASH, has been represented in LoC under the combination of glucose and free fatty acids (usually a 2:1 ratio of oleic and palmitic acid).^6^ Antifibrotic compounds such as obeticholic acid, elafibranor,^7^ pioglitazone, or metformin^8^^,^^9^ showed promising results in reducing lipid droplets in these in vitro settings. Indeed, the anti-NASH agent lanifibranor efficiently reduced hepatocytic lipid accumulation,^10^ and improved human hepatocyte and hepatic stellate cell (HSC) phenotype^11^ in a LoC model but not in 2-dimensional (2D) cell cultures, supporting the specific value of multicellular LoC devices over traditional monocell cultures. Alcohol-associated liver disease has been addressed in several publications, focusing on its impact in sinusoidal cell biology during development^12^ or recovery from alcohol (abstinence) either with perfused spheroid^13^ or layered cultures.^14^ Importantly, Ortega-Prieto et al^15^ developed a model for hepatitis B virus (HBV) long-term infection in primary human hepatocytes that recapitulates virus–host interactions and its associated immune effectors. LoC devices using primary cells isolated through standardized protocols from preclinical models of CLD and from patients' liver tissue also have been developed.^16^^,^^17^ These specialized LoC settings may widen the current knowledge on disease dynamics and provide potential applicability as in vitro preclinical models for drug screening.

Even though LoC complexity has increased outstandingly since the initial models/prototypes, the intricacy of the whole liver still is under-represented. In this regard, several scientists brought the attention and focused their studies on specific processes or structures. For example, the essential features of the bile duct containing primary mouse cholangiocytes,^18^ the unique vasculature organization of the liver,^19^ the sinusoidal zonation within LoC devices,^20^ neutrophil recruitment and interaction with liver sinusoidal endothelial cells (LSECs) after lipopolysaccharide stimulation,^21^ or drug metabolism and toxicity^22^ now are embedded in available LoC systems.

Moreover, CLD has been reported extensively as a systemic syndrome with major extrahepatic implications.^23^ Therefore, the combination of LoC devices now has evolved to the extent that disease-specific models are being combined with others such as intestine, brain, kidney,^24^, ^25^, ^26^ or even metastasis niche-on-chip models^27^ to re-create body-on-chip structures to further study the gut–liver–brain axis, systemic drug clearance, or exosome communication between the liver and the tumor microenvironment. However, alongside these advances in multi-organ and etiology-centered approaches, a lack of consensus in cellular sources and mechanobiological cues within the various LoC models remain as unsolved challenges in the field.

## Liver Scaffolds, Matrices, and Other Substrates

**Figure 3:**
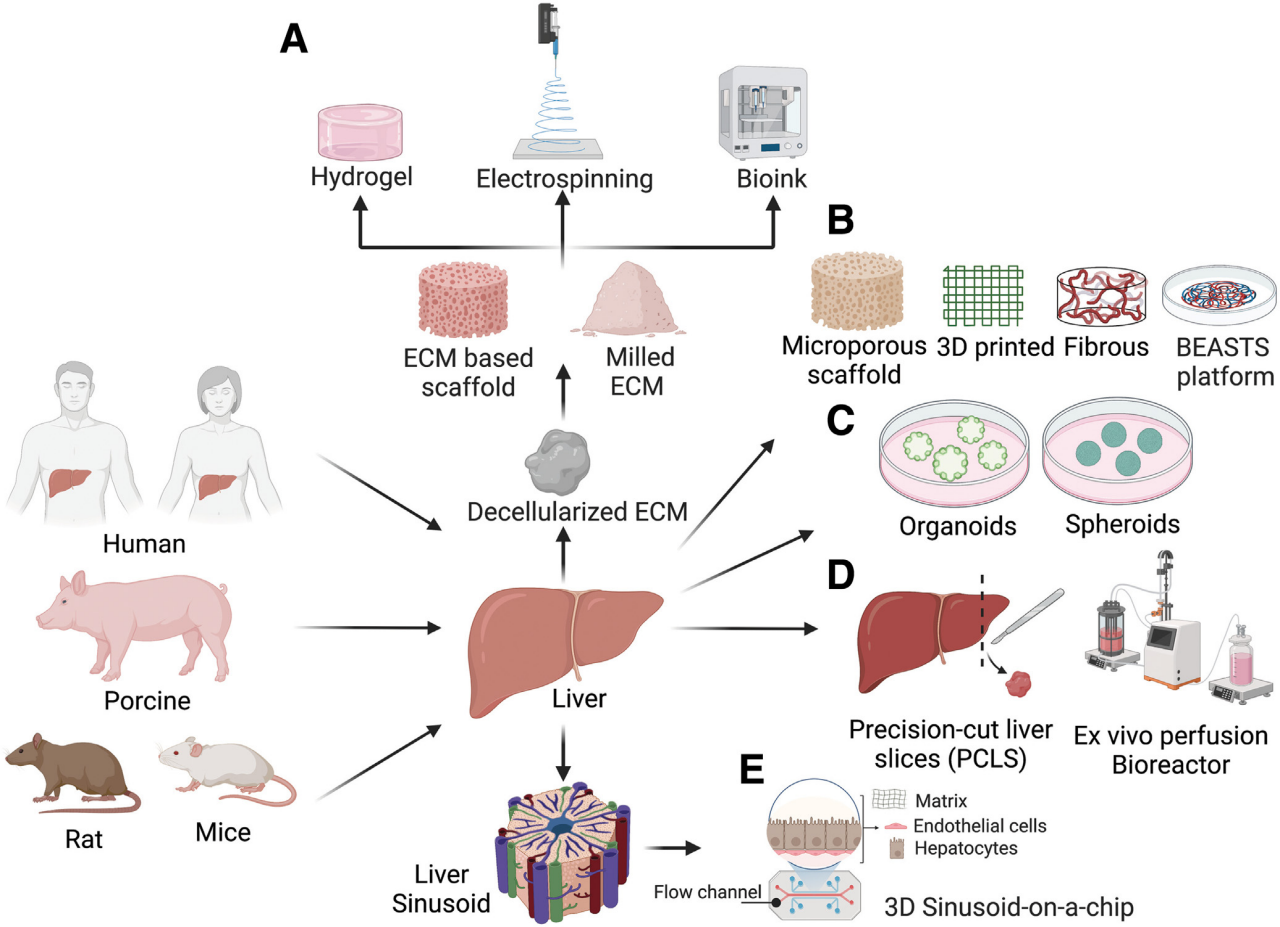
**Schematic overview of in vitro and ex vivo liver models using (*A*) natural scaffolds including hydrogels, fiber-like structures generated by electrospinning, or bioinks, and (*B*) synthetic scaffolds such as microporous, 3D fibrous, or bioengineered platforms to generate (*C*) organoids/spheroids, (*D*) PCLS and bioreactors, and (*E*) 3D liver sinusoid on a chip.** Natural scaffolds obtained from hepatic tissues from various sources such as human, porcine, and rat undergo decellularization using detergents. Synthetic scaffolds including polymer and hydrogel based in combination with novel methods such as 3D printing provides the capability for tuning the properties of the material to re-create the liver microenvironment at stages of disease progression. Current 2D and 3D hepatic models include organoids and spheroids, tissue-based approaches such as PCLS and ex vivo bioreactors, and liver-on-a-chip micropatterned co-culture models. BEASTS, Bio-Engineered Adhesive Siloxane substrate with Tunable Stiffness; ECM, extracellular matrix.

A key component for the engineering of in vitro liver models is the development of the appropriate scaffold/matrix that recapitulates the hepatic microenvironment well enough to result in realistic functional cells. Several factors affect the efficiency of a scaffold as a support for liver cell growth and function including porosity, pore size, biomechanical properties, and the scaffold design. To simulate the microenvironment of natural extracellular matrix (ECM), substrate design and biomechanical properties are of great significance; hence, bioinspired and biomimetic approaches have been explored to model the healthy or damaged liver (Figure 3). Double layers of collagen have been used for years as a well-established 2D in vitro model for sandwich cultures of hepatocytes.^28^ Recent studies have identified, and characterized, the hepatic matrisome comprising ECM signatures beyond collagen that potentially can provide matrix for in vitro systems to study liver diseases.^29^^,^^30^

Scaffolds have been fabricated using natural polymers such as gelatin, elastin, silk fibroin, chitosan, chitin, fibrin, and fibrinogen, or synthetic polymers such as polylactic acid, poly(glycolic acid), polyhydroxyalkanoate, and poly (lactic-co-glycolic acid).^31^ Modified versions of biomaterials such as collagen-incorporated poly (lactic-co-glycolic acid) have resulted in enhancement of hepatocyte survival and functions likely owing to an increase in the bioactivity of the newly developed scaffolds.^32^^,^^33^ Similarly, natural polymers such as silk fibroin have been modified with arginyl-glycyl-aspartic acid (RGD, an integrin-based cell adhesion motif), which has been reported to support the growth of functional hepatocyte clusters.^34^ The modification with RGD also may support attachment of LSECs, known as endothelization of materials.^35^ Efficient spheroid cultures of hepatocytes have been reported on highly porous hydrogel scaffolds composed of alginate and galactosylated chitosan.^36^ In addition, synthetic polymer thin films–based scaffolds allow organized hepatocyte culture and patterned co-culture of hepatocytes with nonparenchymal cells.^37^, ^38^, ^39^ Recent interest in mechanical signaling has led to the development of scaffolds that can re-create liver stiffness in physiological and pathologic conditions. In this context, heparin hydrogel has been developed to modulate stiffness and has shown that hepatocytes cultured on a softer heparin hydrogel (10 kPa) retained 5 times higher levels of albumin production compared with those on a stiffer heparin gel (110 kPa) after 5 days.^40^ Primary hepatocytes grown on modified polyacrylamide gels with cell-adhesive ligands are shown to reduce albumin production and impair hepatocyte function with increasing stiffness.^41^^,^^42^ Desai et al^43^ used polyacrylamide gels to tune the substrate stiffness and showed that fibrotic levels of stiffness significantly inhibit hepatocyte-specific functions in part through the inhibition of the hepatocyte nuclear factor 4α transcriptional network mediated via the Rho/Rho-associated protein kinase pathway. An innovative platform called bio-engineered adhesive siloxane substrate with tunable stiffness based on a polydimethyl siloxane substrate in combination with polyelectrolyte multilayer film-coating technology was developed to engineer mechanically tunable substrates mimicking physiologic and pathologic liver stiffness.^44^, ^45^, ^46^, ^47^, ^48^ More recently, 3-dimensional (3D) bioprinting has emerged for precise spatial positioning of both cells and biomaterials or bioinks such as alginate together in 3D complex geometries and providing mechanical support.^49^^,^^50^ Nguyen et al^51^ have bioprinted hepatocytes and nonparenchymal cells in 3D architecture and developed models of drug-induced liver injury. Recent studies have printed liver cells along with a liver decellularized ECM bioink, creating an environment for maximal cellular function.^52^, ^53^, ^54^ With 3D bioprinting, vascular and biliary fluidic channels also have been created successfully in the LoC device format.^53^ 3D bioprinting of spheroids and organoids represent the next level of technological advancement for creating the highly complex liver architecture.^55^

## Liver Spheroids and Organoids

As described earlier, over the past century, 2D cell cultures have been used as common in vitro models to study cellular responses to stimulation and allowing the construction of low-cost, simple, and well-accepted models of liver disease. However, they do not precisely reflect the true physiological state of cells in vivo owing to the absence of structural, mechanical, and biochemical cues, as well as the interaction between cells and extracellular matrices.^56^ To overcome this limitation, novel 3D cell culture platforms including liver spheroid and organoid cultures are being created to better mimic the in vivo conditions.^57^, ^58^, ^59^, ^60^, ^61^ 3D spheroids are produced via self-assembly, in which monodispersed cells form 3D microtissues called *multicellular spheroids*, and mimic natural processes that occur during embryogenesis, morphogenesis, and organogenesis.^62^ 3D organoids derive from either pluripotent stem cells, neonatal tissue stem cells, or adult stem cells/adult progenitors, in which cells spontaneously self-organize into properly differentiated functional cell types and progenitors, resembling their in vivo counterparts and recapitulating at least some functions of the organ.^63^

In the field of studying liver diseases, recent innovation of hepatic 3D spheroids also offer a promising application via combination of 3D printing–based techniques and HepG2 liver spheroid culture models to develop in situ quantitative evaluation and high-throughput monitoring of drug-induced hepatotoxicity.^64^ HepG2 cell-laden hydrogel constructs were 3D printed in the shape of a cross on the mini-9-well plate, which showed the HepG2 liver spheroids embedded in the gelatin-alginate hydrogel. On the 6th day of culture, HepG2 liver spheroids exposed to varying concentrations of troglitazone and nefazodone were used to predict hepatotoxicity. This model provided a promising tool for screening and characterization of hepatotoxicity in a 3D spheroid-embedded hydrogel system that more closely resembles conditions in vivo.

In 2013, Takebe et al^59^ described the in vitro generation of 3D liver bud organoids from human induced pluripotent stem cell–derived hepatic endoderm cells co-cultured with endothelial and mesenchymal lineages. Interestingly, when these liver buds were ectopically transplanted at various sites including the cranium, subrenal capsule, and distal and proximal-mesentery in immunodeficient mice they were able to rescue the drug-induced lethal liver failure model.^61^^,^^65^ These studies have provided a promising new approach to study regenerative medicine and to translate these techniques for treating patients with end-stage liver failure.^65^ Furthermore, single-cell RNA sequencing (scRNA-seq) data from human liver bud organoids showed several aspects of heterotypic interlineage communication and organ development.^66^ Interestingly, Shinozawa et al^67^ reported a simple, robust, and high-throughput human liver organoid system to measure bile transport activity by live fluorescent imaging with large-scale screening and multiplexed readouts. By using this system, the study analyzed the pathology of drug-induced liver injury and provided the possibility of assessing varying drug susceptibilities based on individual polymorphism at organoid resolution. These approaches are undergoing rapid developments, allowing establishment of human organoids from adult/fetal human liver or pluripotent stem cells and modeling different liver diseases.^68^, ^69^, ^70^, ^71^ Different types of liver organoid models from mice, human beings, dogs, and cats now are available for several monogenic liver diseases such as Alagille syndrome, cystic fibrosis, primary sclerosing cholangitis, Wilsons disease, HBV infection, steatosis, or liver cancer, among others.^72^, ^73^, ^74^, ^75^, ^76^, ^77^, ^78^ The generation of organoids from adult patient liver tissues also retains the genetic background of the individual patient, thus creating patient-specific disease models and enabling in-depth investigations of pathogenesis mechanisms underlying genetic diseases and cancer. In conclusion, induced liver buds and liver organoids provide a platform for cell-based therapy, liver disease models, and drug screening that satisfy the demands of both basic and translational biomedical research.

## Tissue-Based Approaches

Precision-cut liver slices (PCLS) are a native liver–like ex vivo model with intact intercellular and cell-matrix interactions.^79^ PCLS systems use ex vivo liver explants with a well-defined thickness, and, in comparison with the primary hepatocytes that are short-lived and lose much of their function in culture, PCLS cultures have been maintained for 15 days under optimal conditions. Hepatocytes in slices retain their membrane and intracellular polarization, in contrast to isolated hepatocytes, which lose their anatomic polarity after isolation. PCLS cultures have been established both from murine and human livers (Figure 3).

Human tissue for PCLS are obtained from explanted, resected, or nontransplantable tissues from liver tumor patients undergoing transplantation or liver resection. Liver slices also can be obtained from patients with severe fibrosis and cirrhosis undergoing transplantation. These usually are obtained using Krumdieck (now Alabama) tissue slicer to make liver slices with a diametric from 5–8 mm,^79^^,^^80^ and a thickness of 250–350 μm.^81^ These slices then are cultured with William’s E medium in regular tissue plates in either static, dynamic, or bioreactor-based culture systems. In static conditions, PCLS cultures have a shorter lifespan (24–48 hours) resulting from hypoxia and increased cell death. To minimize hypoxic death, strategies such as the use of synthetic oxygen carriers, rocking or shaking cultures, or perfusion bioreactors have been used to provide better perfusion of oxygen and media components.^82^^,^^83^ One study reported PCLS ex vivo cultures with sustained viability for over a 2-week period on a rocking platform.^84^ Through microarray profiling of purified individual cells, this study illustrated that all liver cells undergo changes in their gene expression profiles until day 4 of PCLS cultures, however, these changes seem to be stabilized from day 4 until day 15.

Recently, a study cultured PCLS on a bioreactor platform at a flow rate of approximately 18 μL/s, which imparted functional longevity to the system for approximately 6 days without any hepatocellular stress or fibrogenesis.^83^ Using this system, the study also successfully modeled ex vivo liver fibrogenesis using transforming growth factor β1 and platelet-derived growth factor (PDGF) stimulation. In another similar culture platform, primary hepatocytes or liver stem cells were cultured on ECM discs developed from a decellularized porcine^85^ or rat liver.^86^

Human PCLS cultures have proven indispensable for modeling of liver diseases and to the study of transport, metabolism, and biotransformation of drugs, toxins, and xenobiotics in both normal and diseased conditions.^87^, ^88^, ^89^ They also have been used to study ischemia/reperfusion damage in rodents and to evaluate the efficacy, specificity, and toxicity of virus-mediated gene therapy agents.^80^^,^^90^

With improved technological advancements and culture longevity, PCLS cultures of patient-specific tissues offers enormous potential for the characterization of patient-specific liver cellular heterogeneity and for the screening of novel antifibrotic and antitumorigenic drugs.

## Mimicking the Sinusoidal Mechanobiology In Vitro

LSECs, the second most abundant cell type in the liver, are key players in maintaining hepatic homeostasis.^91^ Importantly, LSECs differ from classic vasculature endothelium because they lack an organized basement membrane and have cytoplasm that is penetrated by open fenestrae, making the hepatic microvascular endothelium discontinuous.^92^ LSEC behavior is largely regulated by shear stress and mechanical stretch induced by blood perfusion and liver microenvironment stiffness changes derived from deposition of ECM.^93^, ^94^, ^95^, ^96^ The effect of these varying mechanical cues on LSECs is particularly interesting, however, this has not been explored extensively until recently. The use of in vitro culture models of LSECs with microfluidic setups showed the effects of shear stress–derived effects. In a pioneering work from the Sessa and Groszmann laboratories, the investigators showed that LSECs respond to increasing shear stress in the microenvironment by increasing nitric oxide (NO) synthesis.^97^ Subsequent work defined the upstream signaling pathways, including the induction of the transcription factor Krüppel-like factor 2, in both healthy and cirrhotic LSECs.^98^ A recent study by Hilscher et al^99^ further elucidated the role of the mechanosensitive pathways in LSECs that drive recruitment of circulating blood cells contributing to portal hypertension and fibrogenesis. Using a flexcell device, cyclic biaxial stretch on murine LSECs was modeled, and showed transcriptional up-regulation of several chemotactic cytokines, neutrophil-extracellular trap activation from the recruited neutrophils, and microthrombi formation contributing to fibrosis. More recently, a LoC device with microfluidics was used to mimic physiological and pathologic pressures on primary LSECs culture.^100^ Transcriptomic analysis showed the detrimental effect of increased pressure on the LSEC phenotype and allowed identification of LSEC-derived pressure-related genes as noninvasive biomarkers for portal hypertension. Altogether these data show that mechanical cues can cause angiocrine and phenotypic changes in LSECs, leading to rapid alteration of HSC phenotype and fibrogenesis.^101^

In fibrotic livers, microvasculature remodeling contributes to increased ECM deposition and the consequent increase in intrahepatic vascular resistance.^92^ Olsen et al^102^ showed that increased stiffness induced activation of HSCs. Juin et al^103^ showed that increased ECM matrix rigidity increased the number of podosomes (actin-rich structures involved in motility and proteolysis) formed in LSECs, suggesting that the cells responded to mechanical stress, however, the effect on LSEC function was not explored. Impairment of hepatocyte and stellate cell function in response to high stiffness has been described previously in the literature.^41^^,^^43^^,^^102^^,^^104^ In the context of liver-specific endothelial cells, a recent publication showed that LSECs also dedifferentiate in high-stiffness conditions, losing their capacity to produce vasoactive mediators such as NO and becoming capillarized as shown by the loss of their characteristic fenestrae. Interestingly, the investigators pointed out the tension between the cytoskeleton and the nuclear shape as a fundamental process transducing the sensing of stiffness into phenotypical responses in all sinusoidal cells.^105^ Importantly, this study also showed that cirrhotic liver cells improve their phenotype when cultured in a healthy (nonstiff) environment, suggesting potential new avenues of therapy development. In an unpublished work, Kidambi et al confirmed that LSECs are responsive to stiffness resulting in rapid capillarization (loss in fenestrae), loss of hyaluronic acid endocytosis, and higher cell adhesion molecules.^46^

These advanced in in vitro experiments point to an interesting and underexplored area of the role of mechanical stimuli on sinusoidal biology during physiological and pathologic conditions. The key to unlocking the potential therapeutic avenues for sinusoidal dysfunction from these in vitro findings will be to integrate the data with in vivo functions.

## “Omics” for the Study of Liver Cells

Advances in omics methods have led to discoveries in liver biology and pathology at the cellular, tissue, and system levels. These methods also have facilitated holistic insight into CLD in the clinical setting, and are generating noninvasive diagnostic modalities for the distinct stages of liver diseases. This multi-omics approach consists of tracing the flow of information from transcriptomics, proteomics, metabolomics, scRNA-seq, single-nucleus analysis, and interactomics. The key findings of these techniques are summarized herein.

Transcriptomics refers to the quantitative assessment of all coding and noncoding RNA transcripts and reflects cellular transcriptional activity. Transcriptomic profiling has resulted in various predictive modalities involving gene expression parameters, targeted measurements, and micro RNA (miRNA) panels with increased functionality in different chronic liver diseases.^106^^,^^107^ Several studies have identified miR-122 as a potential diagnostic biomarker for CLDs. Most of them have shown that miR-122 alone or in combination with other miRNAs (eg, miR-1290, miR-27, miR-192, miR-34, miR-99a) can accurately predict the presence of NAFLD or NASH, but they all perform inadequately when trying to differentiate NAFLD from NASH.^108^, ^109^, ^110^ A recent study performed a comprehensive transcriptomic analysis of primary LSECs during the progression of cirrhosis in which specific molecular signatures, novel biomarkers, and therapeutic targets associated with LSECs dedifferentiation were delineated.^111^

Proteomics refers to the investigation of proteomes—all proteins expressed by a cell. Several studies have investigated the hepatic proteome alone or in combination with the blood proteome, both in animal models or in human beings with CLDs, aiming to answer fundamental pathophysiological questions.^112^^,^^113^ Krahmer et al^112^ assessed the levels and cellular distribution of 6000 liver proteins and 16,000 phosphopeptides in the liver of mice developing hepatic steatosis owing to a high-fat diet. This work produced important fundamental information about the reorganization of organelles, lipid accumulation, and cellular dysfunction that occurs with nutrient overload. Xue et al^113^ identified almost 220 proteins that are significantly different in patients with NAFLD compared with obese metabolically healthy individuals. The proteins that were identified to be increased in CLD were those involved in peroxisome proliferator-activated receptor signaling and ECM receptor interactions whereas the ones that were reduced were localized mainly in mitochondria and involved with oxidative phosphorylation. Expanding on complications of the disease, the proteome of specific cells also has been examined. Vu et al^114^ compared the proteome of hepatocyte monoculture and hepatocytes in organotypic rat liver models, and showed that in a 3D liver model the predominant proteomic phenotype supports fatty acid metabolism and when hepatocytes are cultured in monoculture the proteome shifts to favor glucose metabolism. In addition, they observed an increase in structural and migratory proteins (signaling hepatocyte dedifferentiation) in hepatocyte monoculture, highlighting the need for cell–cell and cell–ECM interactions for maintenance of functional hepatocytes. Lao et al^115^ performed a proteomic analysis between normal and dedifferentiated LSECs. Dedifferentiation and loss of fenestrae in LSECs precedes the onset of fibrosis and is considered a crucial event in the pathology of liver diseases.^92^^,^^116^^,^^117^ A comparison of the normal and dedifferentiated LSECs showed that in dedifferentiated LSECs the most enriched functional categories of proteins were those related to nucleotide, organic acid metabolism, oxidative stress, small molecular and lipid metabolism, cell death regulation, and endocytosis, while those down-regulated by dedifferentiation were transcription regulation, actin cytoskeleton reorganization, cell migration, immune system process, ribosome biogenesis, apoptotic process, angiogenesis, glycerophospholipid metabolism, and cellular lipid metabolism.

Metabolomics refers to the investigation of small molecules and metabolic products, such as amino acids, fatty acids, and carbohydrates. An increasing number of studies have begun to study liver-specific metabolomics in the context of disease using both primary cells and cell culture models. Kim et al^118^ analyzed and compared metabolites in fetal and adult hepatocytes from human donors. They identified 211 metabolites in the hepatocytes. Specifically, the metabolites in the glycolysis/glyconeogenesis pathway, tricarboxylic acid cycle, and urea cycle were lower in fetal hepatocytes than in adult hepatocytes. Li et al^119^ used nuclear magnetic resonance–based metabolomics to investigate the metabolic alterations in hepatocytes caused by HBV infection. They showed that HBV infection contributed to hepatocellular carcinoma (HCC) by up-regulation of the glutamine-fructose-6-phosphate amidotransferase 1-activated hexosamine biosynthesis and choline kinase α-activated phosphatidylcholine biosynthesis. Yue et al^120^ showed using the nuclear magnetic resonance (NMR)-based metabolomic approach that HBV protein (HBx) disrupted the metabolism of glucose, lipids, and amino acids, especially nucleic acids. Min Hae et al^121^ performed metabolic profiling on Huh7 cells with patatin-like phospholipase domain containing 3 (PNPLA3) small interfering RNA silencing and overexpression using gas chromatography–mass spectrometry and liquid chromatography–mass spectrometry metabolic profiling to investigate their role in HCC. Silencing of the PNPLA3 gene resulted in a decrease in amino acid metabolism, suggestive of a catabolic response with extensive protein breakdown. Among the lipids, there was an increase in the levels of myoinositol, cysteine sulfinic acid, polyunsaturated fatty acids, lysolipids, and sphingolipids Overexpression of PNPLA3 mirrored metabolic changes in the opposite direction with an increase in the levels of cholesterol and lactic acid with a shift to anaerobic metabolism. Some of the metabolic signatures associated with the presence of PNPLA3 risk allele such as high cholesterol levels, very low density lipoproteins levels, and so forth, also have been associated with cardiovascular disease in patients with NAFLD.^122^ These and other studies^123^ explain how the use of omics approaches could help to unravel novel phenotype and pathogenesis mechanisms associated with the presence of genetic polymorphisms in complex human liver diseases.

Single-cell, single-nuclei transcriptomics using next-generation transcript sequencing (scRNA-seq/snRNA-seq) is now emerging as a powerful tool to profile cell-to-cell variability on a genomic scale with broad implications for both basic and clinical research.^124^ In a mouse model of liver fibrosis induced by CCl_4_, Krenkel et al^125^ used freshly isolated HSCs for scRNA-seq and found that activation of HSCs and their transdifferentiation toward collagen-secreting myofibroblasts split into heterogeneous populations, characterized by α-smooth muscle actin, collagens, or immunologic markers, while resting HSCs formed a homogenous population characterized by high PDGF-receptor expression. A similar scRNA-seq study using CCl_4_ to induce advanced liver cirrhosis identified 6 clusters of liver endothelial cell (EC) populations including 3 clusters of LSECs that associated with zone-specific transcriptomic changes, 2 clusters of vascular ECs, and 1 cluster of lymphatic ECs.^126^ Hepatotoxicity induced by 2,3,7,8-tetrachlorodibenzo-p-dioxin also showed the diversity of liver cells through the identification of 11 subtypes following pericentral, midzonal, and periportal hepatocyte subpopulations by snRNA-seq, which was a more feasible technique than scRNA-seq in terms of application to frozen samples.^127^ Recently, scRNA-seq was used to characterize mouse embryos on embryonic days 7.5 to 10.5, and Lotto et al^128^ provided a comprehensive atlas of liver cell lineage detailing the divergence of vascular and sinusoidal endothelia, hepatoblast specification, and the emergence of a distinct migratory hepatomesenchymal cell type. The most developed scRNA-seq data set likely is that established by key immune cell populations in the liver, particularly from mouse models of NAFLD/NASH.^129^ A series of elegant studies using scRNA-seq has provided unprecedentedly granular insights into hepatic immune cell heterogeneity, showing striking alterations, particularly in myeloid cells and macrophages in liver diseases,^130^, ^131^, ^132^, ^133^, ^134^ and into related extrahepatic compartments such as bone marrow^135^ or adipose tissue.^136^

Regarding the cellular landscape of the human liver, scRNA-seq also has shown the physiological heterogeneity of human liver cells,^137^^,^^138^ the fibrotic niche of human liver cirrhosis including the identification of pathogenic subpopulations of TREM2+CD9+ macrophages, atypical chemokine receptor 1+, and plasmalemma vesicle–associated protein+ ECs and PDGF-receptor α+ collagen-producing myofibroblasts,^139^ and the immune microenvironment in the context of HCC.^140^ Although scRNA-seq/snRNA-seq remains an expensive and time-consuming technique that requires skilled bioinformatics support, it is a valuable tool to characterize liver function and gene expression dynamics during liver disease, as well as to identify prognostic markers or signatures, and to facilitate discovery of new therapeutic targets.^141^ A key challenge for all mentioned omics techniques is accurate data integration. For instance, the most granular insight into single-cell transcriptomes by scRNA-seq/snRNA-seq techniques comes at the expense of isolating the cells (or nuclei) out of their cellular context^141^; therefore, spatially resolved modalities (eg, multiplex immunostaining, spatial transcriptomics, imaging mass cytometry) are needed to complement these findings.^142^ This has been shown convincingly for immune cell populations in which not only the immune cell phenotype (or single-cell transcriptome), but also their location within the hepatic microenvironment, determines their most likely function during liver diseases.^143^

## Conclusions and Future Directions

As described earlier, in recent years there has been a great advance in the availability and utility of in vitro systems for the study of pathophysiology of the liver. Today, we have a wide range of possibilities to better understand the behavior of cells and tissues in the laboratory, which combine harmoniously with those observations obtained in animal models. Although progress in the field of translational hepatology is evident, we must continue working to create more complete, reliable, and cost-effective systems of human liver diseases. We herein summarize some of the avenues of work that we should develop through collaborative multidisciplinary work, combining the academic and private sectors.

Liver-on-a-chip systems, which already reflect the multicellularity of the liver, should be improved by incorporating biomechanical stimuli typical of the disease under study, such as a specific matrix or sinusoidal pressure, and potentially the relevant immune cells. In addition, the incorporation of biochemical or biological parameter sensors would be of great help for real-time cellular analysis in response to new drugs.

The great potential of 3D liver systems, which currently use mostly matrices of natural origin, has the great advantage of simulating the ECM of the human liver but, at the same time, complicates its standardization and global use. The development and validation of matrices with defined composition, perhaps including the most abundant components in adequate ratios, could assist with expanding their use. Similarly, experimental variables that mimic the biomechanics of the sinusoid (shear stress, pressure, stiffness, and so forth) also should be standardized, thus facilitating the comparison of results from different research groups.

The use of PCLS allows an understanding of the hepatic response to new compounds, but only for a limited period of time. It would be very beneficial to improve the viable incubation time, perhaps by combining several in vitro systems including slices, and the use of tissue from liver disease patients/models.

The field of omics applied to hepatology, and to the rest of biomedical disciplines, is immense and it is difficult to ensure the currency of literature and use of the most advanced techniques. Analysis at the single-cell level, which already is being prototyped using fixed tissue, will transform what we know today as spatial omics. However, tissue cartography requires significant financial investment and excellent experimental design. Therefore, public–private consortiums that include basic scientists and physicians would be of great interest for the sake of advancing knowledge.

Overall, the techniques described in this review and those that are on the horizon can greatly assist to understand liver diseases, develop new therapies, and foster personalized medicine in hepatology. Of course, we need to combine them in a virtuous way, including tissue/cells of human origin whenever possible, and improving the way we mimic human diseases in vitro. If future work is developed further by multidisciplinary teams, success is assured.
